# Preparation and characterization of bear bile-loaded pH sensitive *in-situ* gel eye drops for ocular drug delivery

**DOI:** 10.22038/IJBMS.2020.45386.10562

**Published:** 2020-07

**Authors:** Xiaomin Ni, Qin Guo, Yiqing Zou, Yang Xuan, Imran Shair Mohammad, Qing Ding, Haiyan Hu

**Affiliations:** 1 School of Pharmaceutical Sciences, Sun Yat-sen University, University Town, Guangzhou 510006, PR China; 2 Yunnan Dai Medicine Co., Ltd., Yunnan 678699, PR China

**Keywords:** Bear bile, In-situ ocular gel, pH sensitive, Rheology, Sustain-release

## Abstract

**Objective(s):**

In this study, a stable bear bile-loaded pH sensitive *in-situ* eye drop gel was prepared for sustain delivery and enhanced therapeutic application.

**Materials and Methods:**

Bear bile-loaded *in-situ* ocular gels with different Carbopol/Hydroxypropyl methylcellulose (HPMC) ratios were prepared and their stability was tested in PBS at a series of pH at 40 ^°^C. The morphology was observed by SEM examination and rheology was observed by Rheometer equipped with a 60-mm cone-plate at apex angle of 1^°^. Gel erosion and release kinetics of Tauroursodeoxycholic acid (TUDCA) was determined by HPLC. While, the *in vivo* dwelling time was obtained after administering the fluorescent-loaded gel in ocular disease-free New Zealand rabbits. Finally, biocompatibility and toxicity was observed by irritation test and H&E staining of eye-ball tissues, respectively.

**Results:**

The bear bile-loaded *in-situ* ocular gel showed excellent stability at different pH (pH 5.0, 5.5, 6.0, 6.5, 7.0 and 8.0) up to 5 days, and bear bile extract significantly attenuated the gelling ability of the *in-situ* gel. The viscosity of *in-situ* gels formulation was decreased with increase in shear rate (0.01 to 100 s-1), and morphological examination of freeze-dried preparation showed three-dimensional reticular structure at physiological pH. The *in-situ* ocular gel exhibited promising sustained drug release up to 160 min *in vitro*, and showed prolonged retention time up to 3-folds *in vivo*. Finally, the biocompability data confirmed that the formulation did not induce any toxic effects and was completely compatible with eye tissues.

**Conclusion:**

pH sensitive *in-situ* ocular gel provides new research opportunities to efficiently treat eye diseases.

## Introduction

Ocular diseases not only interrupt vision but also directly affect the quality of life. Between 2000 and 2010, about 2.8 billion of people suffered from visual impairment (1). In this respect, the unique anatomy and physiology of eye contributed to very poor ocular bioavailability of ophthalmic drug delivery systems (2, 3), and limits effective drug concentration in the retina due to the presence of corneal barriers (4). The cornea, composed of epithelium and endothelium with a centered stroma, sabotages the absorption of both lipophilic and hydrophilic drugs. Meanwhile, physiological changes, restricted space of eyeballs, very little instillation volume and short retention time, results in low efficacy and bioavailability of the conventional ocular therapeutics (5). Furthermore, the nictation due external stimuli further suppresses the volume of eye drops. To overcome these problems, various environment-triggered *in-situ* ocular gels, such as ion sensitive, pH sensitive and thermosensitive ocular gels have been widely proposed for ocular drug delivery (6-8). In response of physiological stimuli such *in-situ* gels posed sol-to-gel transition and prolonged the drug retention and release at corneal surface and enhanced the ocular bioavailability (9). On the other side, eyes are very sensitive and cannot tolerate a harsh pH change; therefore, it is quite challenging to choose appropriate gelling materials. In order to achieve pH trigger sol-to-gel transition, the *in-situ* gels must require pharmaceutical excipients in a specific ratio, as phase-transition polymers; such as FDA-approved poloxamer-407 (20% wt.). In contrast, quite a few compounds have been tested as pH sensitive *in-situ* gels formulations for ocular drug delivery including estradiol, levofloxacin and curcumin (10-12). 

Bear bile is a well-known Traditional Chinese Medicine (TCM) and was first documented in Tang Materia Medica (Tang Ben Cao) during the Chinese Tang Dynasty. Previously, the bear bile was widely used in China for more than 3,000 years for the treatment of high-grade fever in children, infantile convulsion and eye redness, demonstrating its promising therapeutic potentials (13, 14). Importantly, bear bile has been employed to treat visual disorders, such as glaucoma, retinitis pigmentosa, protection of photoreceptor and age-related macular degeneration. Furthermore, Tauroursodeoxycholic acid (TUDCA), the major component of bear bile, showed promising therapeutic outcomes in retinal ganglion, light-induced retinal degeneration, retinal ganglion cell death following optic nerve crush, preventing lens epithelial cell (LEC) death, cataract formation, controlling apoptosis in age-related macular degeneration and retinitis pigmentosa (15-17). 

**Scheme 1:**
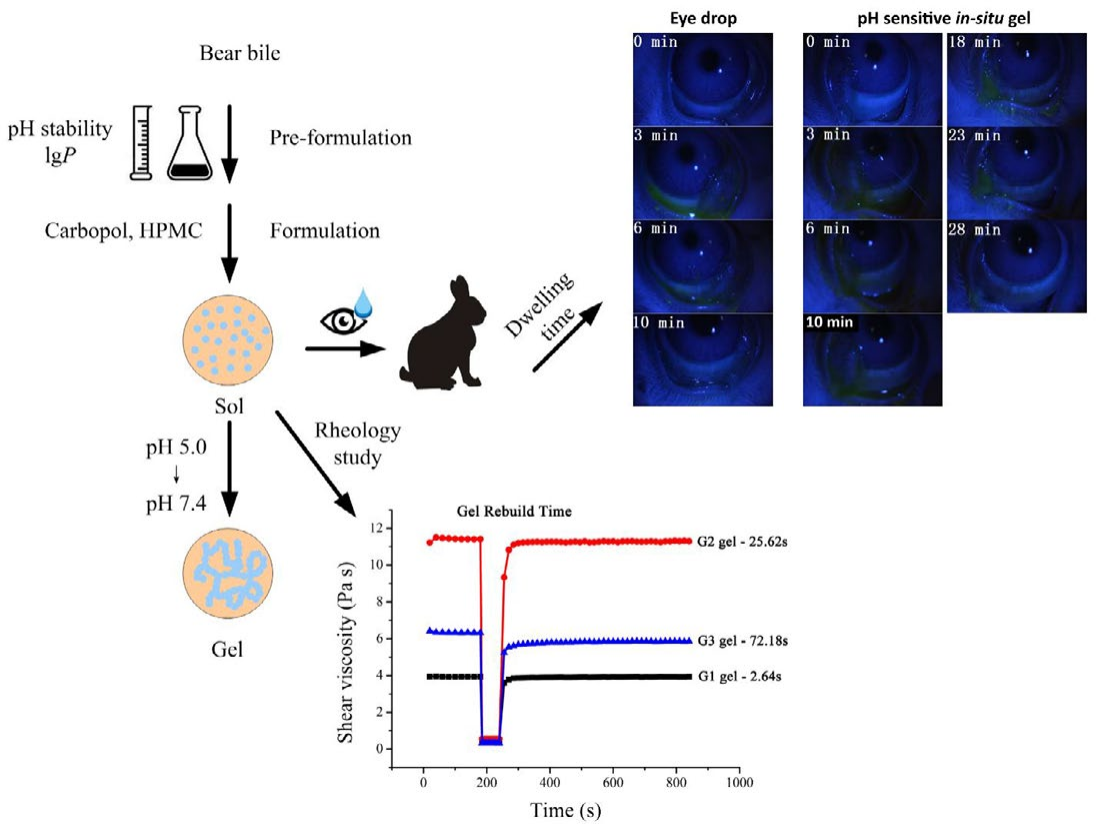
The preparation method, sol-gel phase transition, rheology and dwelling time to evaluate *in vitro* and *in vivo* effect of bear bile-loaded pH sensitive *in-situ* gel

However, the present bear bile ophthalmic formulations showed limited therapeutic efficacy due to various anatomical and physiological factors. Moreover, the precorneal-drainage of eyes further required its repeated administration. Equally important, the stability and penetrability variances of drugs to cornea within a dramatic pH range need to be investigated. In view of this, we aimed to develop a bear bile-loaded pH sensitive *in-situ* gel formulation by using a mixture of Carbopol-974 and the Hydroxypropylmethylcellulose (HPMC) K4M is a high purity, water-soluble cellulose derivative polymers to enhance the therapeutic efficacy of bear-bile, as describe in scheme 1. 

## Materials and Methods

***Materials***

Bear bile and bear bile eye drops were provided by Yunnan Dai Medicine Co., Ltd. (Yunnan, China). Sodium tauroursodeoxycholate (TUDCA) was obtained from National Institutes for Food and Drug Control (Beijing, China). Carbopol^®^-974 was purchased from Lubrizol (OH, USA) and HPMC-K4M was supplied by The Dow Chemical Company (MI, USA). All reagents were of analytical grade.

***Animals***

Ocular disease-free New Zealand white rabbits (2 kg) were obtained from Guangdong Medical Lab Animal Center (Foshan, China) and housed individually at Laboratory Animal Center of Sun Yat-Sen University (Guangzhou, China) with free access to water and food. All the animal experiments were approved by Animal Ethical and Welfare Committee of Sun Yat-sen University.

***Effects of bear bile on the gelling capacity of Carbopol/HPMC***

To determine the effects of bear bile on the gelling capacity of polymers, Carbopol-974 and HPMC-K4M were dispersed in deionized water for complete swelling. Next, bear bile and deionized water were introduced to obtain drug-loaded *in-situ* gel (0.3% Carbopol and 0.5% HPMC). Blank *in-situ* gel was prepared following similar procedure except the incorporation of bear bile. Triethanolamine (20%) was used to adjust gels to a series of pH. Viscosity of the gels was determined by a rotational rheometer (Malven Kinexus, Worcestershire, UK) and plotted against pH.

***Preparation of bear bile-loaded pH sensitive in-situ ocular gels***

Bear bile-loaded *in-situ* ocular gels with different Carbopol/HPMC ratios (G1, G2, G3) were prepared. Briefly, Carbopol-974 and HPMC-K4M were dispersed in deionized water and allowed to swell at 4 ^°^C. After the swelling process, a mixture of ethyl paraben, ethylenediaminetetraacetic acid (EDTA) and mannitol, and bear bile were added. Finally, triethanolamine (20%) was used to adjust the pH (5.0±0.1) of bear bile-loaded formulation. The complete composition of all bear bile-loaded pH sensitive *in-situ* ocular gels formulation is given in Table 1.

***Stability assessment of bear bile at different pH***

The stability of different *in-situ* ocular gel formulations was tested in a series of pH phosphate buffered solutions (pH 5.0, 5.5, 6.0, 6.5, 7.0 and 8.0) at 40 ^°^C (18). The samples were taken on day 0, 1, 3 and 5, followed by filtration with 0.45 μm millipore filter. Next, 15 μl filtrate was assayed for TUDCA (major component of bear bile) by HPLC equipped with a C-18 column (Waters X-Bridge, 250 x 4.6 mm, 5 μm) using mobile phase (methanol/0.03 mol/l monosodium phosphate, 65:35, v/v, pH 4.4) at 35 ^°^C with flow rate of 1 ml/min. Furthermore, to determine partition coefficient of bear bile, bear bile was diluted with n-octanol-saturated at different pH (pH 5.5, 6.0, 6.5, 7.0 and 8.0). The diluted samples (3 ml) were then added to PBS saturated with n-octanol solution (3 ml). After shaking in a thermostatic oscillator (34 ^°^C) for 72 hr, the mixtures were centrifuged at 3000 g (10 min). Next, the aqueous phases were collected and instantaneously assayed for TUDCA by HPLC. The Papp (apparent oil/water partition coefficient) was calculated as:

Papp = (*C*_0_-*C*_w_)/*C*_w_

Where, *C*_0_ was the drug concentration in aqueous phase before partition,* C*_w_ was the drug concentration in aqueous phase after partition and *C*_0_-*C*_w_ was the drug concentration in oil phase after partition.

***Morphological examination***

The morphology of *in-situ* gels was observed by SEM examination (JSM-6330F, JEOL, Japan) at pH 5.0 and pH 7.2. Briefly, a suitable amount of *in-situ* gels (G1, G2 and G3) was separately placed on a watch glass and freeze-dried for 2 days. Next, the freeze-dried samples with intact surface were mounted on a copper plate and gold sprayed before being photographed by SEM. 

***Rheological examination***

Rheological behaviors were determined by Rheometer equipped with a 60-mm cone-plate at apex angle of 1^°^. The shear viscosity measurements of the *in-situ* gels were performed at 25 ^°^C by changing the shear rate from 0.01 to 100/sec. Shear modulus including elasticity and loss was determined between 0.01 Hz and 20 Hz with strain of 0.05% (which was pre-determined by strain scanning at 1 Hz in the range of 0.1% to 50%). The thixotropy was observed by recording the recovery time of shear viscosity, when the gel was subject to steep change in shear rate.

***In vitro*** ***gel erosion and drug release kinetics***

To determine *In vitro* gel erosion, the *in-situ* gels G1, G2 and G3 were loaded in respected vials and adjusted the pH (7.2) with triethanolamine (20%) to make complete gelatinization. Then, 2 ml of artificial tear at 34°C was added under shaking at 100 g for next 20 min. Next, the dissolution media were immediately removed and the vials were weighted again. The procedure was repeated until less than 10% of initial *in-situ* gel was left in each vial. The cumulative gel erosion was plotted against time. Next, the TUDCA release was investigated by dissolution followed by HPLC method. Briefly, pH sensitive* in-situ* ocular gel formulations (G1, G2, G3) were placed in small glass bottle with cap in release medium (pH 7.2) and allowed to form gel. Next, 2 ml of simulated artificial tear was added and placed in an incubator with a shaking speed of 100 rpm/min at 37 ^°^C. At pre-determined time points, 1 ml of sample was withdrawn and replenished with the fresh release medium. Upon centrifugation at 15,000 rpm for 5 min and filtration through 0.45 μm membrane, the concentrations of TUDCA in the supernatant were detected by HPLC system equipped with Waters X-Bridge C-18 column (250x4.6mm, 5 µm) at 210 nm. A mixture of methanol and sodium dihydrogen phosphate (65:35, v/v) and water (80: 20, v/v) was used as the mobile phase at a flow rate of 1 ml/min at 37 ^°^C. The injection volume was 15 μl. Finally, Higuchi equation, non- and monoexponential functions were applied to explain the mechanisms of erosion and *in vitro* drug release in Origin 8.0 software.

***Determination of*** ***in vivo*** ***dwelling time of optimized formulation***

The *in vivo* dwelling time of *in-situ* ocular gel was determined on New Zealand white rabbits. Briefly, 40 μl of formulation containing 0.05% of sodium fluorescein was instilled in the conjunctival sac of the left eye (treatment group). While, the same volume of commercially available bear bile eye drop containing the same content of sodium fluorescein was dropped in the right eye (control group). The eyes of all rabbits were then closed deliberately for 6 to 10 sec. Next, the cornea and conjunctival sac were observed for fluorescence by a slit lamp and then photographed and the time just before the disappearance of fluorescence was recorded. 

***In vivo*** ***biocompatibility***

The biocompatibility of *in-situ* ocular gel was observed by irritation test. Briefly, 40 μl of a single-dose formulation was administrated to the left and the same volume of normal saline was dropped into the right eyes of the rabbits, respectively (n=3) for a period of 2 weeks. The irritation of the eyes was scored according to Draize technique (19). Finally, all animals were sacrificed after final observation and eyeballs were removed for H&E staining and observed for any sign of toxicity.

***Statistical analysis***

All results are presented as means±SD, and each value was the mean of three replicate independent experiments performed in parallel. Statistical analyses were performed using un-paired t-test. The statistical significant difference is mentioned in figures legend.

## Results

***Effects of bear bile on the gelling capacity of Carbopol/HPMC***

**Figure 1:**
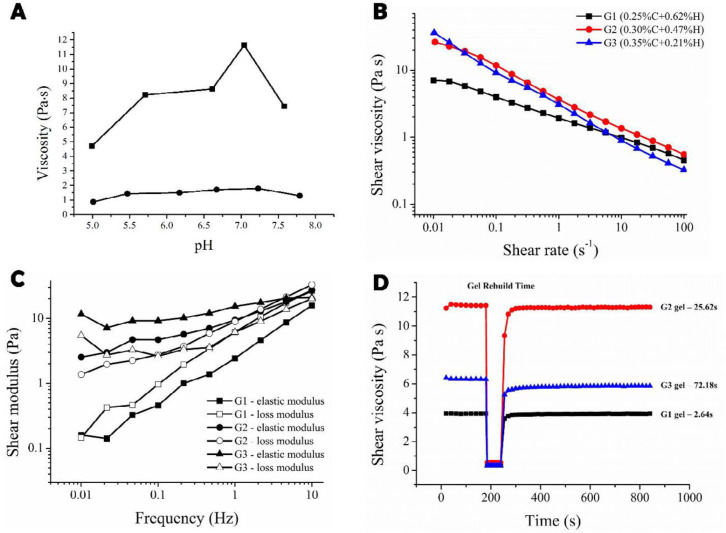
(A) The change of the gelling capacity caused by bear bile. : Blank gel matrix; :Gel matrix treated with bear bile. Rheology studies of the bear bile-loaded pH-sensitive *in-situ* ocular gels. (B) Viscosity of the *in-situ* gels in response to shear rate for G1 (black), G2 (red) and G3 (blue). (C) Frequency scanning results in viscoelasticity test for G1 (elastic modulus G’, loss modulus G’’); G2 ( elastic modulus G’, ○loss modulus G’’), and G3 (elastic modulus G’, loss modulus G’’). (D) Thixotropy test results for G1 (black), G2 (red) and G3 (blue)

The effect of bear bile on gelling capacity of Carbopol/HPMC was determined and the results showed that after addition of bear bile, the pH sensitivity of the *in-situ* gel was almost eliminated, suggesting bear bile extract significantly attenuated the gelling ability of Carbopol/HPMC. Furthermore, a rapid change and a zenith of viscosity was observed at relatively narrow pH range of 6.4 to 7.7 Figure (1A), perhaps due to the presence of Ca^2+^, Mg^2+^, and Zn^2+^ in bear bile (20). Notably, the reduction of bear bile-loaded gel sensitivity to pH, and variation of viscosity with the loading content due to bear bile composition, we selected pH 5.0 as the initial pH for the pH sensitive *in-situ* ocular gels. Moreover, these observations also suggested that the role of bear bile cannot be neglected; therefore, we adjusted the amount of excipient to develop *in-situ* gel formulations. 

***Preparation and stability assessment of bear bile-loaded pH sensitive in-situ ocular gels***

Bear bile-loaded *in-situ* ocular gels with different Carbopol/HPMC ratios (G1, G2, G3) were prepared by dispersing respective amount of Carbopol-974 and HPMC-K4M in deionized water and allowed to swell at 4 ^°^C. Then, a mixture of ethyl paraben, EDTA and mannitol, and bear bile was added at pH 5.0. The complete composition of all bear bile-loaded pH sensitive *in-situ* ocular gel formulations is given in Table 1. Notably, the metabolism of drugs occurs via chemical reactions as it contact with enzymes. Thus, *in vitro* stability and partition coefficient was determined and the results showed that TUDCA, the major component of bear bile, at different pH remained unchanged, indicating good stability of bear bile at different pH (Table 2). 

On the other side, the partition coefficient of TUDCA in octanol/water system increased up to pH 0.2 to 0.3 at pH 5.0-8.0, respectively (Table 3). 

***Rheological examination***

The rheological evaluation showed that the viscosity of G1, G2 and G3 formulations decreased as shear rate increased from 0.01 to 100 s-1 Figure (1B)**,** exhibiting pseudoplastic fluid with typical shear thinning. Therefore, when applied, the *in-situ* gels would reduce viscosity under the shearing force caused by winking reflex and easily spread across the eye surface (21). Importantly, the G2 and G3 gels showed more shear-thinning than G1 as observed from steeper slopes of curves. For viscoelasticity assay, both elasticity modulus (G’) and loss modulus (G’’) were frequency-dependent and increased with frequency in all three tested gels, Figure (1C). In contrast, the G1 had the lowest G’ and G’’ with G’’>G’, which exhibited lower gel strength. Comparatively, G’-G’’ intersections existed in G2 and G3’s curves over the frequency range, where G’ was predominant and indicated higher gel strength followed by G’’, which helped to resist the shear force by winking reflex. The G3 had a higher gel strength than G2 as reflected by the larger G’ at all tested frequencies. Finally, to determine thixotropic behavior of *in-situ* gel, gel rebuild time after withdrawn of the shear force was recorded for each gel Figure (1D). The G1 took the shortest time (2.64 sec) to recover, while the gel rebuild time for G3 was over 1 min (72.18 sec). G2 gel had a moderate gel rebuild time (25.62 sec). Thus, gel rebuild times of G1, G2 and G3 probably increase with concentration of the Carbopol (22). 

***Morphological examination***

**Figure 2:**
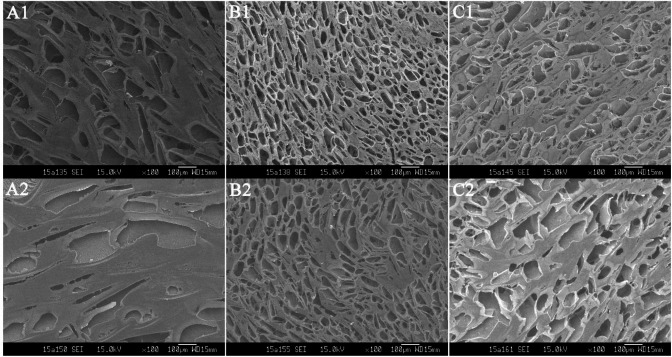
The morphology of bear bile-loaded pH-sensitive *in-situ* ocular gels in initial and physiological pH conditions. (A1): G1, pH 5.0; (A2): G1, pH 7.2; (B1): G2, pH 5.0; (B2): G2, pH 7.2; (C1): G3, pH 5.0; (C2): G3, pH 7.2

The morphological examination of freeze-dried *in-situ* gels showed three-dimensional reticular structure at physiological pH, Figure 2. All the gels showed pores of different sizes, shapes and depths. Notably, the morphology of G2 was much denser and more porous than the G1 and G3 formulations (Figure S-1). Whereas, the depths of pores in all gels were decreased at physiological pH than at its initial pH, indicating that the polymer swelled in response to increase in pH (23), which further confirmed sol-to-gel transition. Surprisingly, the microscopic observations found that all tested gel formulations possessed 3-D porous network structures, which contributes to the controlled release of loaded drug (24).

***In vitro*** ***gel erosion and release kinetics***

**Figure 3:**
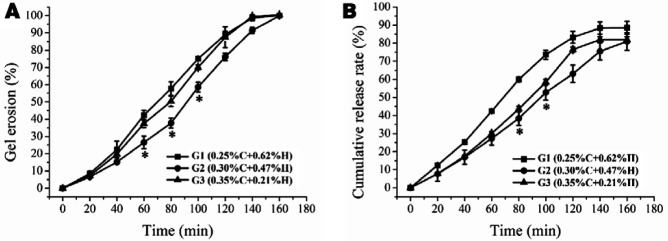
Gel erosion and drug release behaviors of the bear bile-loaded pH-sensitive *in-situ* ocular gels. (A) Cumulative erosion curves of pH-sensitive bear bile-loaded *in-situ* ocular gels. G1 (), G2(), G3 (). (B) Cumulative release profiles of bear bile-loaded pH-sensitive *in-situ* ocular gels G1 (), G2 (), G3(), where *P<0.05

The prolonged dwelling time of ocular gels required not only to resist the shear force as stated above but also moderate thixotropy to rebuild, quickly after the shear force disappeared. In contrast, the short gel rebuild time prevents *in-situ* gel from being washed away by tear, which significantly contributes to its extended dwelling time. *In vitro* gel erosion and TUDCA release of *in-situ* gels was monitored up to 160 min. Compared to G1 and G3 formulation, the G2 formulation exhibited significantly slow erosion at 60, 80, 100 min, while showed sustained release specifically at 80, 100 min, as depicted in Figure (3A, B). 

As expected, eyewink and lacrimal secretion of the eyes resulted in thinning and erosion of the *in-situ* gel. Consequently, the drug release on eye surfaces did not follow Fick’s law of diffusion and the release kinetics was depended on the erosion behaviors of the carrier matrix. Consistently, the fast erosion speed resulted in the fast drug release, and vice versa (25). This gel erosion and drug release kinetics could also be related with microstructures of *in-situ* gels. Table 4 provides a summary of the fittings of different kinetics models of gel erosion and drug release for *in-situ* gels. It was observed that G1 was fitted to Higuchi model (R2=0.9843), in which the erosion rate was decreased with time. Whereas, G2 and G3 gels exhibited zero-order erosion kinetics (R2=0.9849; R2=0.9771) indicating a constant erosion rate. In release behavior, the G1 gel followed first-order kinetics (R2=0.9789), while gels G2 and G3 followed either zero-order (G2: R2=0.9937; G3: R2=0.9727) or first-order release kinetics (G2: R2=0.9931; G3: R2=0.9709).

***Determination of*** ***in vivo*** ***dwelling time***

**Figure 4:**
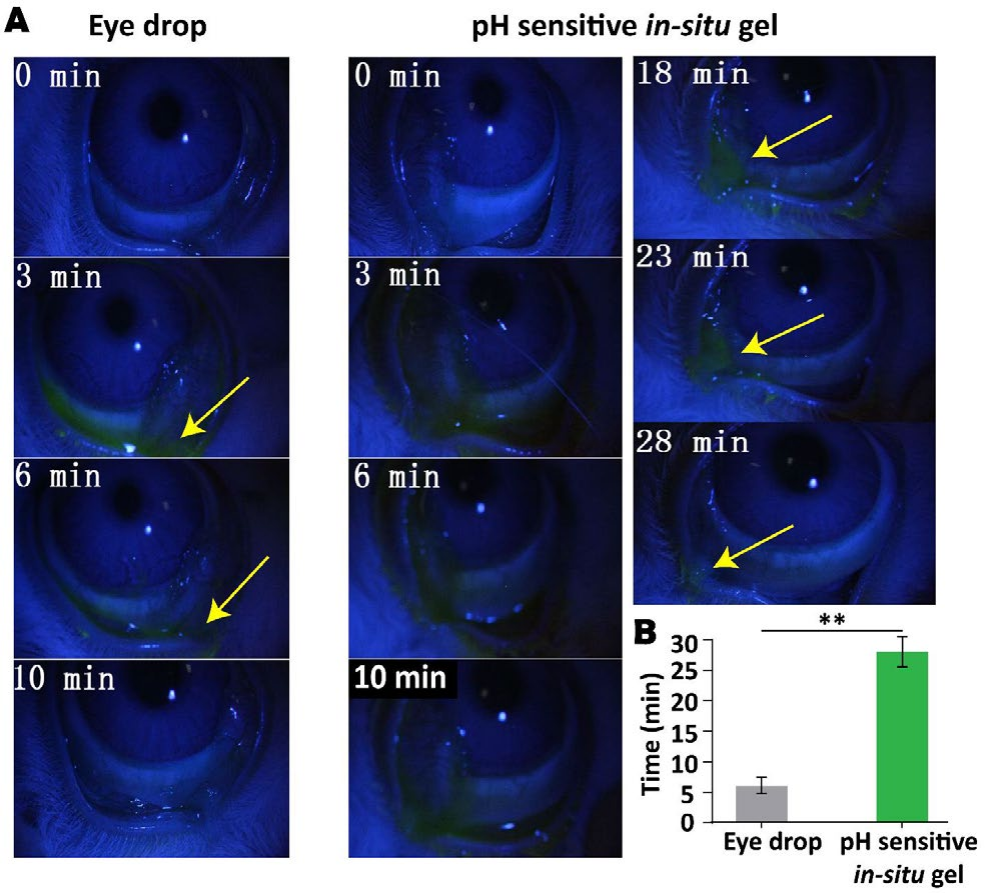
*In vivo* ocular retention time of commercially available bear bile eye drops and G2 formulation in ocular disease free New Zealand white rabbits (n=3) , where **P<0.01

The dwelling time of ocular gel formulation was determined by administering fluorescence-gel formulations. In eye drop group, the fluorescence was mainly accumulated in conjunctival sac just after instillation. The majority of drops were quickly drained to canthus and nasolacrimal duct due to frequent blinking and tear turnover, and the fluorescence was disappeared after 9 min. In contrast, the retention time of G2 in conjunctival sac significantly prolonged up to 28 min Figure (4A, B), which contributed to a prolonged contact time with corneal tissue to ensure improved therapeutic efficacy. 

***In vivo*** ***biocompatibility***

**Figure 5.**
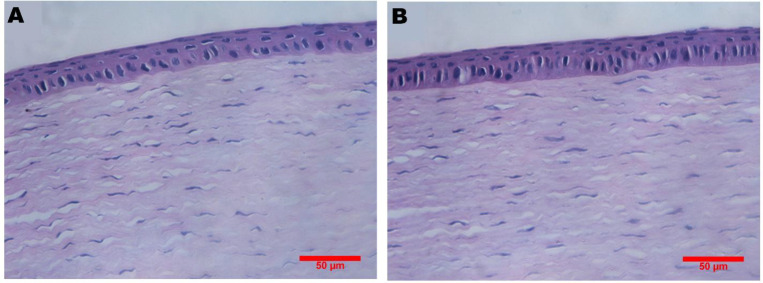
Representative corneal pathological sections of rabbits; A: treated with saline; B: treated with G2 formulation

The *in vivo* compatibility examination showed that neither single nor multiple-dosing administration caused any abnormities in cornea, iris or conjunctiva of rabbits. Importantly, the corneal H&E sections of eyeballs post-treatment with *in-situ *gel did not show any significant difference, as compared to control Figure 5, confirming that gel was non-irritant and completely biocompatible.

**Table 1:** Composition of pH sensitive bear bile-loaded *in-situ* ocular gels (g in 100 g)

**Formulation**	**Bear bile extract**	**Carbopol-974**	**HPMC-K4M**	**Ethyl paraben**	**EDTA**	**Mannitol**
G1	10.0	0.25	0.62	0.06	0.01	4.50
G2	10.0	0.30	0.47	0.06	0.01	4.50
G3	10.0	0.35	0.21	0.06	0.01	4.50

**Table 2:** Drug content (%) determination of Tauroursodeoxycholic acid in buffer solution at different pH

	**pH**					
**Time (d)**	**5.0**	**5.5**	**6.0**	**6.5**	**7.0**	**8.0**
0	100.0	100.0	100.0	100.0	100.0	100.0
1	99.7	99.0	98.7	99.2	99.1	99.0
3	100.0	102.8	102.2	102.8	102.8	102.5
5	99.8	97.6	104.2	103.7	103.1	103.3

**Table 3:** Partition coefficient of Tauroursodeoxycholic acid in octanol/water

**pH**	**C**_0_	**C**_w_	**Papp**	***log*** ***P***
5.5	1.2265	0.9864	0.2428	-0.6147
6.0	1.2248	0.9762	0.2536	-0.5958
6.5	1.2612	0.9905	0.2750	-0.5606
7.0	1.2539	0.9599	0.3069	-0.5130
8.0	1.2632	0.9149	0.3822	-0.4177

**Table 4:** Gel erosion and drug release kinetics of the *in-situ* ocular gels

**Formulation**		**Gel erosion**																
		**Zero-order equation** (y=a+b*t)						**First-order equation** (y=a*(1-exp(-b*t))							**Higuchi equation** (y=a+b*t ^1/2^ **)**			
		**a**		**b**		**R** ^2^		**a**			**b**	**R** ^2^			**a**		**b**	**R** ^2^
G1		-0.02		0.007		0.97		4.50			0.001	0.97			-0.51		0.12	**0.98**
G2		-0.13		0.007		0.98		1938.47			3E^-6^	0.95			-0.6		0.12	0.94
G3		-0.06		0.007		0.98		233.79			3E^-5^	0.97			-0.56		0.12	0.97
**Formulation**		**Drug release**															
		**Zero-order equation** (y=a+b*t)						**First-order equation** (y=a*(1-exp(-b*t))					**Higuchi equation** (y=a+b*t ^1/2^ **)**				
		**a**		**b**		**R** ^2^		**a**	**b**			**R** ^2^	**a**		**b**		**R** ^2^
G1		0.04		0.006		0.95		1.65	0.0053			0.98	-0.35		0.10		0.97
G2		-0.02		0.005		0.99		-2.94	-0.0016			0.99	-0.41		0.09		0.97
G3		-0.02		0.005		0.97		29.12	0.0002			0.97	-0.44		0.10		0.96

## Discussion

Derived from gallbladder of black bear, bear bile has long been used as a valuable TCM. Its unique and major component, TUDCA showed promising results in the treatment of refractory eye diseases including glaucoma, and retinal macular degeneration (26). Besides various cholates, bear bile also contained amino acids, fats, phospholipids and trace metals (27). Furthermore, bear bile eye drops are available in market and have been approved for treating acute or chronic catarrhal conjunctivitis. Despite its prominent therapeutic potentials in ophthalmology, the repeated administration due to precorneal drainage leads to poor compliance and reduced therapeutic efficacy. Furthermore, various known and unknown components of TCM may affect the gelling capacity of polymers. The incorporation of bear bile into the gel matrix significantly reduced the sensitivity of gel to pH, suggesting that the role of drug cannot be neglected in ophthalmic formulations, thus we enabled the gelling capacity by adjusting the amounts of excipients (Figure 1). 

For pH-triggered *in-situ* gels, their initial pH (pH 5.0) significantly depends on its composition (9). However, eyes can tolerate a relatively narrow range of pH (pH 4.8 to 9.0), thus it is quite challenging to choose appropriate gelling materials (28). In this respect, the stable formulation was obtained by attenuating the amount of carbopol combined with HPMC (Table 1). Intriguingly, their excellent stability at various tested pH (acidic or basic) makes them a promising material for ocular drug delivery system (Table 2). On the other side, low *log P* values implied increased hydrophilicity of bear bile, which limits its absorption through conjunctiva and cornea (29). Fortunately, this could prolong the dwelling time of *in-situ* gel (Figure 3). In addition, its partition coefficient remained unchanged within a relatively wide range of pH (Table 3), suggesting that variation in pH does not affect corneal drug absorption. 

The rheological parameters, shear viscosity and viscoelasticity determine physical stability and applicability of ophthalmic gels (30). During blinking, shear rate ranges from 4,250 to 28,500 s-1; therefore, gels with shear thinning would be suitable for ocular application, i.e., low viscosity at high shear rate and high viscosity at low shear rate (pseudoplastic fluid). Although the viscosity of optimized G2 formulation was comparatively high, but it was in liquid state; therefore, it accurately instilled and uniformly covered the eyes surface. Due to protective mechanism of eye, the conventional ophthalmic formulations showed very low bioavailability (<5%) and short duration of therapeutic action (31). Thus, prolonged dwelling time of ocular gels requires not only suitable strength to resist the shear force as stated above but also moderate thixotropy to rebuild the gel quickly. The G2 formulation demonstrated reasonable rebuilt time than G1 and G3, contributes to improved dwelling time, protecting from being washed by tears. Intriguingly, all the formulations (G1, G2, G3) followed non-Newtonian flow and had shear thinning properties. They, however, differed in viscoelasticity and thixotropy with G2 formulation having suitable viscoelasticity and less thixotropy (rebuilt time: 25.62 s), which were well adaptable with ocular physiological behaviors.

The pH dependent sol-to-gel transition directly reflects by variations in depth of pores (32), where denser pores indicated slow gel-erosion and drug release. This gel erosion and release kinetics were also related with the microstructures of the gels. Surprisingly, the microscopic observations found that all tested gel formulations possessed 3-D porous network structures, which were particularly favorable for controlled release of drug payload (Figure 2). The changes in structure of gel before and after gelation represent variations in pores depth, where the denser pores of G2 indicated slow gel erosion and drug release (Figure 3). Finally, the irritation reactions account largely for poor-compliance or incompatibility of ophthalmic drug delivery systems and represent an important factor for ocular gel assessments (33). In *in vivo* biocompatibility assessment, the bear bile-loaded *in-situ* gel showed no acute or chronic pathological signs and was completely compatible with the eyes (Figure 4, 5). 

## Conclusion

We developed a stable bear bile-loaded pH sensitive *in-situ* ocular gel for sustain drug delivery and characterized by taking full consideration of the ocular physiological factors, *in vitro* and *in vivo.* The different tested pH did not influence the stability and *logP* of TUDCA. While, rheological parameters of pH sensitive *in-situ* ocular gels were in accordance with the retention time of drug and the gels resistance capacity, against the physiological reactions of eyes. Besides, less thixotropy indicated shorter rebuild time and quick recovery, which were indeed helpful to prevent the *in-situ* gel from being cleared by tear and prolonged the contact time with corneal tissue to ensure improved therapeutic efficacy. Furthermore, compared with marked eye drops, the *in-situ* ocular gels displayed an extended *in vivo* dwelling time and sustained drug release, along with complete biocompatibility with eye tissues. Finally, the newly developed pH sensitive *in-situ* gel not only provides guidelines for the preparation and characterization of pH sensitive *in-situ* ocular gels but also offer new research opportunities for TCM drug delivery for eye diseases. 
